# Hemoglobin predicts mortality risk in geriatric patients with dysphagia: a Japanese retrospective cohort study

**DOI:** 10.3389/fnut.2026.1746775

**Published:** 2026-06-16

**Authors:** Ping Yuan, Li Zhuo, Jingyi Xue, Xinglin Gao

**Affiliations:** 1 Department of General Section I, Guangdong Provincial Geriatrics Institute, Guangdong Provincial People’s Hospital, Guangdong Academy of Medical Sciences, Southern Medical University, Guangzhou, China; 2 Division I, Department of Geriatric Respiratory, Guangdong Provincial Geriatrics Institute, Guangdong Provincial People’s Hospital, Guangdong Academy of Medical Sciences, Southern Medical University, Guangzhou, China

**Keywords:** anemia, dysphagia, elderly, hemoglobin, mortality, percutaneous endoscopic gastrostomy

## Abstract

**Background:**

Hemoglobin (Hb) is an established prognostic biomarker in several conditions, but its prognostic value in elderly patients with dysphagia is unclear.

**Objective:**

To evaluate whether Hb concentration predicts all-cause mortality in Japanese geriatric patients with dysphagia.

**Methods:**

We performed a retrospective secondary analysis of a publicly available Japanese cohort (Dryad; *n* = 253; Jan 2014–Jan 2017). Hemoglobin measured ≤7 days before nutritional support initiation was analyzed as a continuous variable and by tertiles (T1 < 9 g/dL; T2 9–12 g/dL; T3 ≥ 12 g/dL). Cox proportional hazards models estimated associations (Models I–III: unadjusted to fully adjusted). Survival probabilities were assessed by Kaplan–Meier analysis; subgroup interactions were tested.

**Results:**

Median age was 83 years (*n* = 253; 154 females). In multivariable analysis Hb (per 1 g/dL) was independently associated with lower mortality (adjusted HR = 0.83; 95% CI: 0.76–0.91; *p* < 0.001). Compared with T1, adjusted HRs were 0.62 (95% CI: 0.41–0.93; *p* = 0.021) for T2 and 0.36 (95% CI: 0.21–0.60; *p* < 0.001) for T3. Median survival increased across tertiles (T1:185 days; T2:309 days; T3:405 days; log-rank *p* < 0.001). No significant subgroup interactions were observed.

**Conclusion:**

Higher hemoglobin concentration was independently associated with lower mortality risk in this Japanese cohort of hospitalized geriatric patients with advanced dysphagia requiring artificial nutrition, supporting routine Hb monitoring to inform nutritional and prognostic care.

## 1 Introduction

Dysphagia—clinically defined as impaired deglutition (1)—is designated by the WHO as a high-impact disorder contributing to excess morbidity, mortality, and healthcare costs (2). Globally affecting 590 million individuals (3), this underdiagnosed yet prevalent condition demonstrates progressive age-dependent incidence (4), fulfilling diagnostic criteria for a geriatric syndrome with 10–33% prevalence in elderly populations (5) and 11–60% among community-dwelling seniors (6). Swallowing is a complex neuro-muscular process at which around 50 muscle couples need to be coordinated. The swallowing process is controlled by the swallowing centers in cortex and brainstem as well as by five brain nerves. Severe dysphagia appears most frequently in the context of neurological illnesses (7), notably stroke, Parkinson’s disease, and dementia (8). Specifically, stroke patients exhibit dysphagia rates of 37–78% (9), while >80% of Alzheimer’s disease and 60–80% of Parkinson’s disease cases develop swallowing impairment during disease progression (10, 11). Iatrogenic dysphagia occurs in 70–80% of nasopharyngeal carcinoma patients post-radiotherapy and 1–79% following anterior cervical fusion (12). Crucially, multivariate analyses establish dysphagia as an independent mortality predictor (13) that elevates risks for malnutrition, aspiration pneumonia, dehydration, doubled 6-month mortality (14), 1.7-fold increased mortality (15), and long-term care institutionalization (5). This evidence base mandates implementation of standardized screening algorithms and early targeted interventions.

Anemia is an independent risk factor for decreased health-related quality of life in older individuals (16). It is associated with all-cause mortality (17), as well as mortality due to CVD (18, 19), cancer, and respiratory disease (20). Anemia frequently accompanies percutaneous endoscopic gastrostomy (PEG), correlating with reduced survival and serving as a metabolic distress indicator portending poor prognosis (21). Notably, it predicts 4-week mortality in PEG-dependent nutritional support patients (22). Post-stroke rehabilitation data further associate low baseline hemoglobin as a significant risk factor for sarcopenia, delayed functional recovery, and dysphagia progression (23). Optimal gastrointestinal management prioritizes symptom control while monitoring complications, particularly anemia and malabsorption (24). The prognostic role of hemoglobin concentrations in mortality risk stratification for geriatric dysphagia patients represents a significant and clinically relevant research gap, warranting further investigation given its potential utility as an accessible prognostic indicator.

Despite these clinical implications, standardized guidelines for geriatric dysphagia management remain inadequate. Early assessment is consequently essential for adverse outcome prognostication. Although hemoglobin demonstrates prognostic value across multiple pathologies, its relationship with mortality in dysphagia patient’s especially Japanese elderly is poorly defined. With mortality predictors in this population lacking consensus, this study specifically examined hemoglobin’s independent correlation with mortality in Japanese geriatric patients with dysphagia.

## 2 Materials and methods

### 2.1 Data source

The present study is a secondary analysis of a de-identified dataset deposited in the Dryad Digital Repository (25). The original study was conducted at Miyanomori Memorial Hospital and details of data collection, inclusion/exclusion criteria and primary ethical approvals are reported in the source publication. We accessed and analyzed the publicly available dataset in compliance with Dryad terms and the ethical framework described in the primary report. The present secondary analysis did not involve direct contact with human participants.

### 2.2 Study design and participants

We conducted a retrospective cohort analysis at a single institution involving dysphagia patients aged ≥65 years who received percutaneous endoscopic gastrostomy (PEG) or total parenteral nutrition (TPN) from January 2014 to January 2017. Multidisciplinary clinical assessments (physicians, nurses, speech-language pathologists) combined with video fluoroscopy confirmed severe dysphagia in all cases. Exclusion criteria encompassed: (1) terminal malignancies; (2) PEG for gastric decompression; (3) PEG placement prior to January 2014. The Miyanomori Memorial Hospital Ethics Board approved this study and waived informed consent due to anonymized retrospective data.

### 2.3 Procedures

Nutritional support modality (PEG vs. TPN) was determined through shared decision-making between clinicians and patients (or their family members). Appropriate nutrition was administered based on the clinical evaluations conducted by healthcare professionals. Clinical information, including age, sex, underlying diseases such as cerebrovascular diseases, severe dementia, aspiration pneumonia, ischemic heart disease (IHD), the presence of non-tunneled central venous catheters (NT. CVC), percutaneous endoscopic gastrostomy (PEG), oral intake recovery, and laboratory parameters were obtained from patients’ medical records. Hemoglobin values were measured ≤7 days before initiating nutritional interventions. The primary objective of this study was to assess mortality rates following the initiation of the procedure during the designated follow-up period. Participants were stratified into three groups (T1 < 9 g/dL, T2 9–12 g/dL, T3 ≥ 12 g/dL), based on their hemoglobin at the time of enrollment.

### 2.4 Statistical analysis

Publicly available datasets enabled secondary analyses.

Baseline characteristics are presented as mean ± standard deviation (SD) for normally distributed continuous variables, median (interquartile range, IQR) for skewed continuous variables, and counts (percentages) for categorical variables. Between-group comparisons used one-way ANOVA or Kruskal–Wallis tests for continuous variables and chi-square or Fisher’s exact tests for categorical variables, as appropriate. Survival time was defined as days from initiation of nutritional support (date of PEG insertion or start of TPN) to death or last follow-up. Hemoglobin (Hb) was analyzed both as a continuous variable (per 1 g/dL increase) and categorically by prespecified groups (T1: <9 g/dL; T2: 9–12 g/dL; T3: ≥12 g/dL). We fitted Cox proportional hazards models to estimate hazard ratios (HRs) and 95% confidence intervals (CIs). Model I was unadjusted; Model II adjusted for age and sex plus major comorbidities (cerebrovascular disease, severe dementia, aspiration pneumonia, ischemic heart disease); Model III additionally adjusted for markers of inflammation and treatment factors (CRP, NT-CVC, PORT, PICC, PEG, oral intake recovery and daily kcal). Subgroup analyses examined effect modification by prespecified variables using likelihood ratio tests for interaction; *p*-values for interaction are reported. Kaplan–Meier curves and log-rank tests compared survival across Hb categories; median survival times with 95% CIs are reported. All tests were two-sided and *p* < 0.05 was considered statistically significant. Analyses were performed in Free Statistics 2.2.

## 3 Results

### 3.1 Participant characteristics

The cohort comprised 253 patients (99 males, 154 females) with baseline characteristics detailed in Table 1. Mean age was 83.1 ± 9.3 years. Nutrition support included PEG (*n* = 180) and TPN (*n* = 73). Significant intergroup differences (*p* < 0.05) emerged for age, ischemic heart disease, PEG utilization, NT-CVC presence, CRP levels, and hemoglobin concentrations.

**Table 1:** Baseline characteristics of patients.

Variables	Total	Hb, T1 (<9)	Hb, T2 (9 ~ 12)	Hb, T3 (≥12)	*p*
	(*n* = 253)	(*n* = 46)	(*n* = 120)	(*n* = 87)	
Age(year)	83.1 ± 9.3	85.2 ± 6.7	84.5 ± 7.6	80.0 ± 11.7	< 0.001
Sex, *n* (%)					0.534
Male	99 (39.1)	16 (34.8)	45 (37.5)	38 (43.7)	
Female	154 (60.9)	30 (65.2)	75 (62.5)	49 (56.3)	
CI, *n* (%)	133 (52.6)	25 (54.3)	56 (46.7)	52 (59.8)	0.17
Dement, *n* (%)	102 (40.3)	23 (50)	52 (43.3)	27 (31)	0.069
Asp., *n* (%)	94 (37.2)	16 (34.8)	50 (41.7)	28 (32.2)	0.354
IHD, *n* (%)	47 (18.6)	16 (34.8)	21 (17.5)	10 (11.5)	0.004
CRP (mg/dl)	1.0 (0.3, 3.3)	2.1 (0.6, 5.8)	1.3 (0.5, 4.0)	0.4 (0.1, 1.5)	< 0.001
Hb(g/dl)	11.0 ± 2.0	7.9 ± 0.9	10.6 ± 0.8	13.1 ± 0.9	< 0.001
PEG, *n* (%)	180 (71.1)	26 (56.5)	82 (68.3)	72 (82.8)	0.004
NT. CVC, *n* (%)	73 (28.9)	20 (43.5)	38 (31.7)	15 (17.2)	0.004
Oral, *n* (%)	15 (5.9)	0 (0)	10 (8.3)	5 (5.7)	0.094
Status, *n* (%)					< 0.001
Alive	115 (45.5)	8 (17.4)	49 (40.8)	58 (66.7)	
Dead	138 (54.5)	38 (82.6)	71 (59.2)	29 (33.3)	

CI, cerebrovascular diseases; Dement, severe dementia; Asp., aspiration pneumonia; IHD, ischemic heart disease; NT. CVC, non-tunneled central venous catheters; PEG, percutaneous endoscopic gastrostomy; Oral, oral intake recovery; CRP, C-reactive protein; Hb, Hemoglobin.

### 3.2 Kaplan–Meier curve

**Figure 1:**
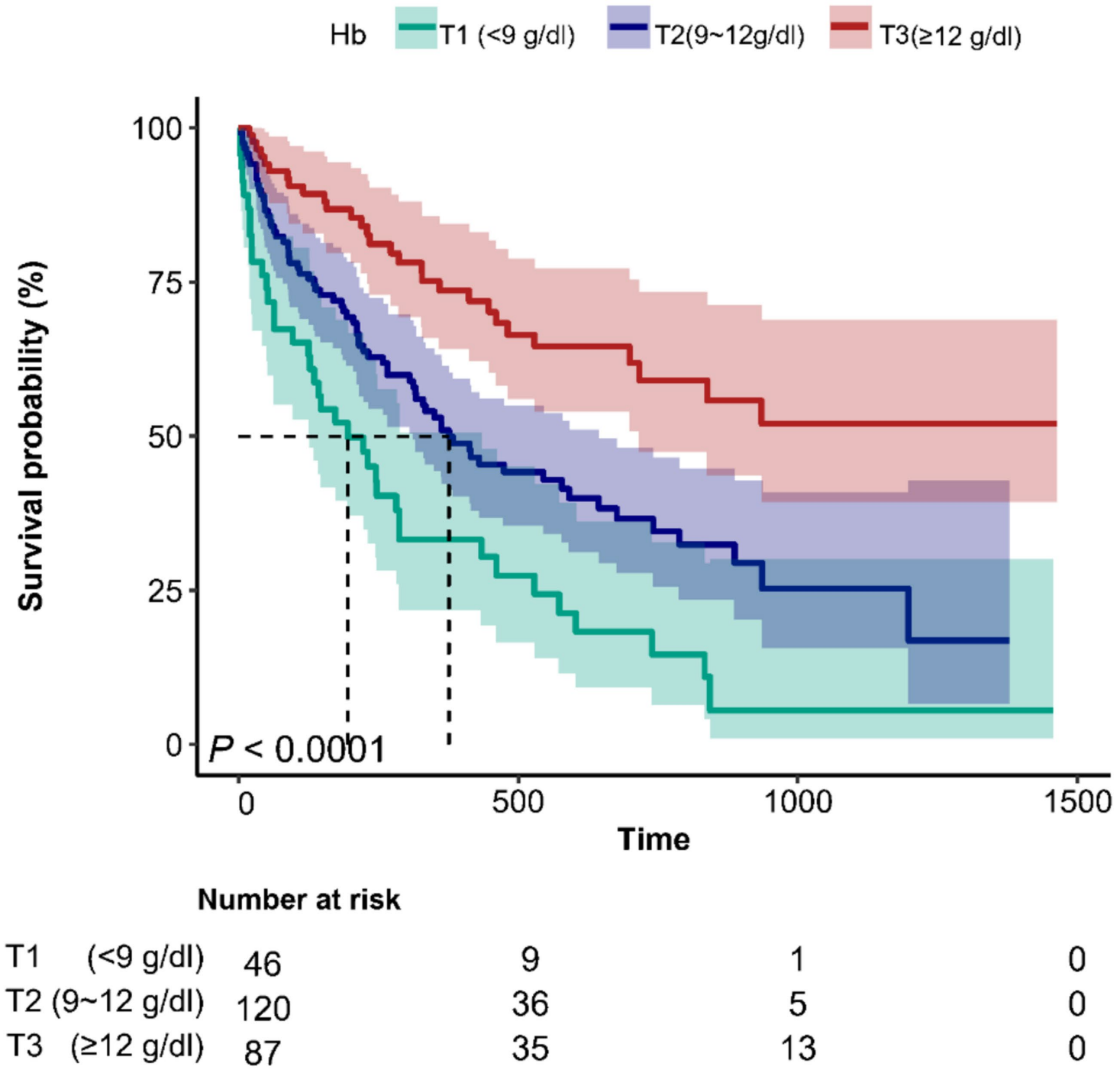
Kaplan–Meier survival analysis for mortality with hemoglobin in three groups.

Figure 1 displays Kaplan–Meier survival curves. T2 and T3 cohorts exhibited substantially prolonged median survival versus T1 (309 and 405 days vs. 185 days; log-rank *p* < 0.001).

### 3.3 Association between hemoglobin and mortality in various models

Table 2 illustrates the hazard ratio (HR) and 95% confidence intervals (95% Cl) associated with the risk of mortality in patients with dysphagia based on hemoglobin. There are four models for hemoglobin as continuous and categorical variables. No variables to be chosen to adjust in model I. Variables of demographics (age, sex) and past medical history (cerebrovascular diseases, severe dementia, aspiration pneumonia, IHD) were chosen to adjust in model II. All the variables that we included (age, sex, cerebrovascular diseases, severe dementia, aspiration pneumonia, IHD, C-reactive protein, non-tunneled central venous catheters, implantable central venous ports, peripherally inserted central catheters, percutaneous endoscopic gastrostomy, oral intake recovery and Kcal/day) to be adjusted in model III. The results of these models were shown in Table 2. The risk of mortality exhibited an downward trend as hemoglobin increased in the univariable Cox regression analysis (HR = 0.77, 95% CI: 0.71–0.84, *p* < 0.001). Upon adjusting for all covariates in the multivariable Cox regression analysis, the HR was 0.83 (95% CI: 0.76–0.91, *p* < 0.001). When compared to the lowest hemoglobin group (T1 < 9 g/dL), the adjusted HR values for hemoglobin and mortality in the T2 (9–12 g/dL) and T3 (≥ 12 g/dL) groups were 0.62(95% CI: 0.41–0.93, *p* < 0.05) and 0.36 (95% CI: 0.21–0.6, *p* for trend < 0.001), respectively.

**Table 2:** Association between hemoglobin and mortality in different models.

Variable	Model I		Model II		Model III	
	HR (95% CI)	*p*	HR (95% CI)	*p*	HR (95% CI)	*p*
Hb	0.77 (0.71 ~ 0.84)	<0.001	0.79 (0.73 ~ 0.86)	<0.001	0.83 (0.76 ~ 0.91)	<0.001
Hb (T1,< 9 g/dL)	1(Ref)		1(Ref)		1(Ref)	
Hb (T2,9–12 g/dL)	0.55 (0.37 ~ 0.82)	0.003	0.55 (0.37 ~ 0.81)	0.003	0.62 (0.41 ~ 0.93)	0.021
Hb (T3,≥12 g/dL)	0.25 (0.15 ~ 0.41)	<0.001	0.31 (0.19 ~ 0.51)	<0.001	0.36 (0.21 ~ 0.6)	<0.001
*p* for Trend		<0.001		<0.001		<0.001

Model I: non-adjusted. Model II: adjusted for age, sex, cerebrovascular diseases, severe dementia, aspiration pneumonia, ischemic heart disease. Model III: adjusted for age, sex, cerebrovascular diseases, severe dementia, aspiration pneumonia, ischemic heart disease (IHD), C-reactive protein (CRP), non-tunneled central venous catheters (NT-CVC), implantable central venous ports (PORT), peripherally inserted central catheters (PICC), percutaneous endoscopic gastrostomy (PEG), oral intake recovery and Kcal/day.

### 3.4 Subgroup analyses

**Figure 2:**
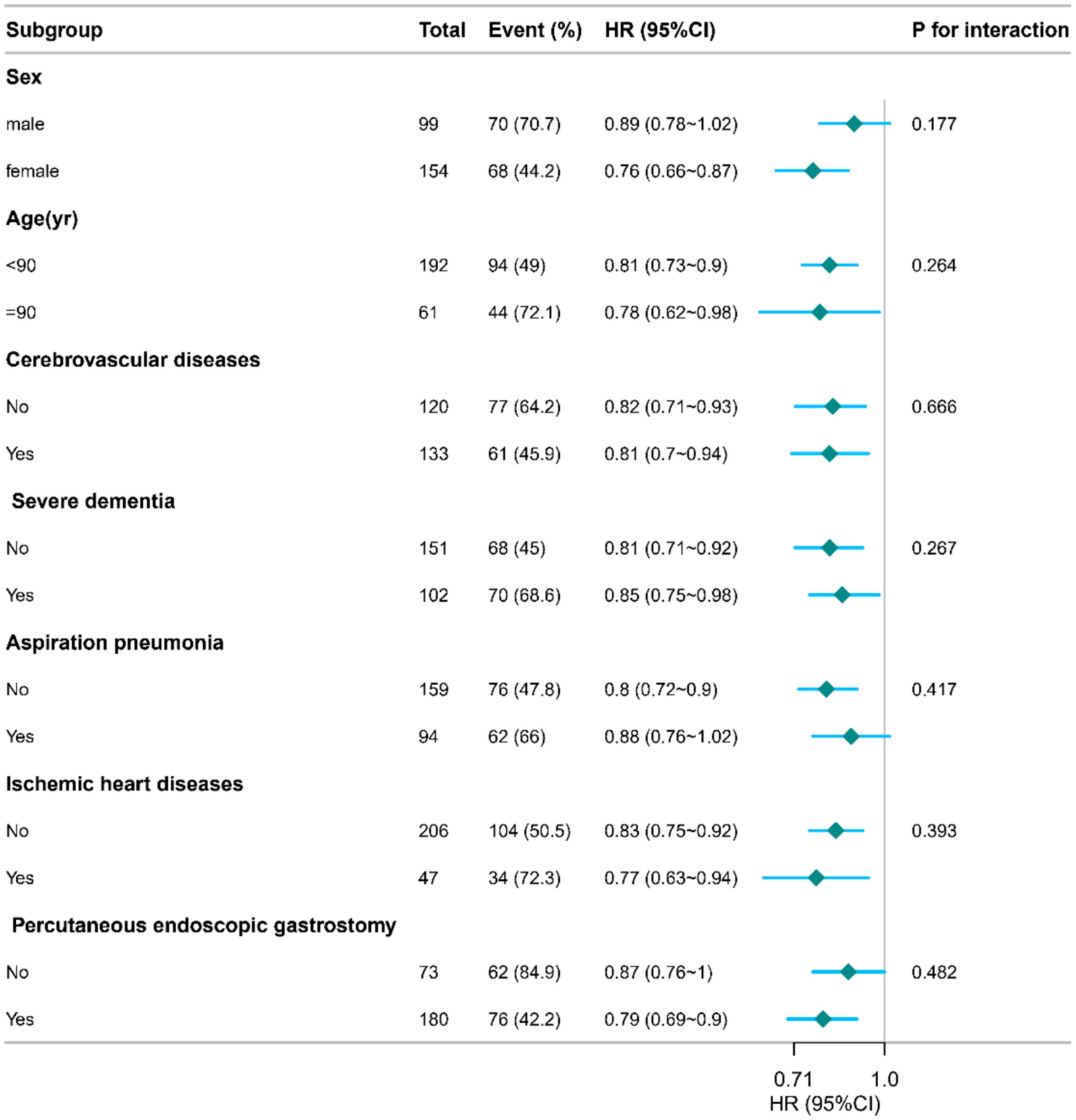
Subgroup analyses of hemoglobin associated with mortality. Hazard ratios (HRs) were adjusted for age, sex, IHD, cerebrovascular diseases, severe dementia, aspiration pneumonia, PEG, CRP, PORT, NT-CVC, PICC, Kcal/day, oral intake recovery.

Figure 2 presents interaction analyses after comprehensive adjustment for: age, sex, IHD, cerebrovascular disorders, severe dementia, aspiration pneumonia, PEG, CRP, PORT, NT-CVC, PICC, daily caloric intake, and oral recovery status. No significant interactions emerged across seven subgroups, confirming result robustness.

## 4 Discussion

This study found that Hb concentration was independently associated with lower all-cause mortality in a Japanese cohort of hospitalized geriatric patients with advanced dysphagia receiving artificial nutrition. When modeled as a continuous variable, a significant inverse Hb–mortality relationship was observed in unadjusted analyses (HR = 0.77, 95% CI 0.71–0.84, *p* ≤ 0.001) and persisted after adjustment for covariates (adjusted HR = 0.83, 95% CI 0.76–0.91, *p* ≤ 0.001). Categorical analysis by Hb tertile yielded a stepwise decline in mortality: multivariable-adjusted models indicated 38% lower risk for T2 versus T1 and 64% lower risk for T3 versus T1 (both *p* ≤ 0.05).

The primary focus of this study was Hb, regarded as a marker of nutritional status in patients with dysphagia. Malnutrition substantially contributes to the elevated mortality observed in older patients with dysphagia. Nutritional deficits impair immune competence by compromising immune cell function and the host’s ability to control infection and regulate inflammatory responses. Malnutrition also increases oxidative stress and the production of pro-inflammatory mediators, promotes tissue injury and perturbs the gastrointestinal microbiota, all of which can exacerbate disease severity. Accordingly, prevention and management of geriatric dysphagia should prioritize optimization of nutritional status to support immune function and tissue integrity, reduce inflammation, and ultimately mitigate mortality. It should be noted, however, that nutritional supplementation by enteral or intravenous routes carries an infection risk that must be balanced against potential benefits.

The recent guideline emphasizes prompt assessment and management of dysphagia to prevent adverse effects on quality of life, notably dehydration, malnutrition and aspiration pneumonia. It emphasizes the importance of promptly evaluating and addressing dysphagia to prevent negative impacts on an individual’s quality of life. These impacts include dehydration, malnutrition, and aspiration pneumonia (26). Furthermore, researches (27, 28) indicated that more than half of dysphagia patients experience complications such as malnutrition and dehydration due to eating disorders, which worsen the progression of the disease.

These results are biologically and clinically coherent in the context of dysphagia. Geriatric patients with dysphagia are at high risk of malnutrition and its downstream consequences—sarcopenia, immune compromise and functional decline—which in turn are associated with excess mortality (9, 15, 27, 28), Stroke-related dysphagia, for example, increases the risk of aspiration pneumonia and nutritional deterioration, and patients requiring enteral feeding frequently present with anemia of chronic disease or multifactorial anemia (21). Accordingly, dysphagia in Geriatric adults requires systematic intervention: early nutritional screening, routine monitoring for anemia and other complications, timely initiation of targeted nutritional and rehabilitation strategies, and multidisciplinary care pathways to mitigate downstream morbidity and mortality (29). In this setting lower Hb may therefore reflect greater physiological disturbance and worse prognosis rather than representing a direct causal driver of mortality, that confirming routine Hb monitoring to improve risk stratification and clinical vigilance.

Clinically, our findings support the role of Hb as a pragmatic prognostic marker for risk stratification in older patients with dysphagia. Routine Hb surveillance may aid early identification of patients at elevated risk and prompt further assessment—nutritional evaluation, investigation of anemia etiology, and consideration of targeted nutritional and rehabilitative input. Finally, Hb measurement is widely available, inexpensive and scalable, which makes it an attractive candidate for incorporation into dysphagia care pathways pending prospective validation. Future work should focus on testing whether incorporation of Hb into multimodal prognostic models improves risk prediction and whether targeted interventions informed by such monitoring affect patient-centered outcomes.

This study identified hemoglobin concentration as an independent, inverse predictor of mortality in patients with dysphagia (30), supporting its role as a pragmatic prognostic marker in clinical practice. Prioritizing improvement of nutritional status is therefore essential to reduce mortality in this vulnerable population. Routine hemoglobin surveillance can facilitate early recognition of clinical deterioration and trigger timely interventions (nutritional optimization, diagnostic evaluation for causes of anemia, or targeted rehabilitation). With global population aging and increasing dysphagia incidence after stroke (31, 32) and certain orthopedic procedures hip fracture (33), anterior cervical fusion (34), hemoglobin measurement—readily available, low-cost, and routinely performed—offers a scalable, cost-effective element for mortality risk stratification.

Nevertheless, several limitations warrant consideration. First, the study was conducted at a single center in Japan and was retrospective, potentially compromising its accuracy compared to more robust multicenter prospective studies from different countries. Second, the cohort is restricted to severe dysphagia requiring artificial nutrition, which limits applicability to: Mild/moderate dysphagia, Community-dwelling elderly. Third, we must recognize the potential presence of selection bias in our study due to the reliance on a solitary measurement of hemoglobin within a 7-day timeframe upon hospitalization without subsequent follow-up measurements. Consequently, the potential effect of hemoglobin levels at varying time intervals on the desired outcomes was precluded. Finally, as a secondary analysis of publicly available data, the investigators could not influence study design, data collection, variable definitions. Together, these limitations underscore the need for prospective, multicenter studies with repeated hemoglobin measurements and standardized data collection to validate and extend the present findings.

## 5 Conclusion

Higher hemoglobin concentration was independently associated with lower mortality risk in this Japanese cohort of hospitalized geriatric patients with advanced dysphagia requiring artificial nutrition. Subgroup analyses demonstrated no significant interaction effects, confirming the association was robust across the clinical subgroups examined. Taken together, these findings support the potential utility of hemoglobin as a low-cost prognostic biomarker in the management of advance geriatric advance dysphagia.

## Data Availability

The original contributions presented in the study are included in the article/supplementary material, further inquiries can be directed to the corresponding author.
